# HTLV-1 modulates the frequency and phenotype of FoxP3^+^CD4^+^ T cells in virus-infected individuals

**DOI:** 10.1186/1742-4690-9-46

**Published:** 2012-05-30

**Authors:** Yorifumi Satou, Atae Utsunomiya, Junko Tanabe, Masanori Nakagawa, Kisato Nosaka, Masao Matsuoka

**Affiliations:** 1 Laboratory of Virus Control, Institute for Virus Research, Kyoto University, Kyoto, 606-8507, Japan; 2 Department of Hematology, Imamura Bun-in Hospital, Kagoshima, 890-0064, Japan; 3 Department of Neurology, Graduate School of Medical Science, Kyoto Prefectural University of Medicine, Kyoto, 602-8566, Japan; 4 Department of Hematology, Kumamoto University School of Medicine, Kumamoto, 860-8556, Japan; 5 Current address: Immunology Section, Division of Infectious Diseases, Department of Medicine, Imperial College, London, W2 1PG, UK

**Keywords:** HTLV-1, ATL, HAM/TSP, FoxP3, Tax, HBZ

## Abstract

**Background:**

HTLV-1 utilizes CD4 T cells as the main host cell and maintains the proviral load via clonal proliferation of infected CD4^+^ T cells. Infection of CD4^+^ T cells by HTLV-1 is therefore thought to play a pivotal role in HTLV-1-related pathogenicity, including leukemia/lymphoma of CD4^+^ T cells and chronic inflammatory diseases. Recently, it has been reported that a proportion of HTLV-1 infected CD4^+^ T cells express FoxP3, a master molecule of regulatory T cells. However, crucial questions remain unanswered on the relationship between HTLV-1 infection and FoxP3 expression.

**Results:**

To investigate the effect of HTLV-1 infection on CD4^+^ T-cell subsets, we used flow cytometry to analyze the T-cell phenotype and HTLV-1 infection in peripheral mononuclear cells (PBMCs) of four groups of subjects, including 23 HTLV-1-infected asymptomatic carriers (AC), 10 patients with HTLV-1 associated myelopathy/tropical spastic paraparesis (HAM/TSP), 10 patients with adult T-cell leukemia (ATL), and 10 healthy donors. The frequency of FoxP3^+^ cells in CD4^+^ T cells in AC with high proviral load and patients with HAM/TSP or ATL was higher than that in uninfected individuals. The proviral load was positively correlated with the percentage of CD4^+^ T cells that were FoxP3^+^. The CD4^+^FoxP3^+^ T cells, themselves, were frequently infected with HTLV-1. We conclude that FoxP3^+^ T- cells are disproportionately infected with HTLV-1 during chronic infection. We next focused on PBMCs of HAM/TSP patients. The expression levels of the T_reg_ associated molecules CTLA-4 and GITR were decreased in CD4^+^FoxP3^+^ T cells. Further we characterized FoxP3^+^CD4^+^ T-cell subsets by staining CD45RA and FoxP3, which revealed an increase in CD45RA^−^FoxP3^low^ non-suppressive T-cells. These findings can reconcile the inflammatory phenotype of HAM/TSP with the observed increase in frequency of FoxP3^+^ cells. Finally, we analyzed ATL cells and observed not only a high frequency of FoxP3 expression but also wide variation in FoxP3 expression level among individual cases.

**Conclusions:**

HTLV-1 infection induces an abnormal frequency and phenotype of FoxP3^+^CD4^+^ T cells.

## Background

Human T-cell leukemia virus type 1 (HTLV-1) is a delta type retrovirus, which causes leukemia of HTLV-1-infected CD4^+^ T cells, known as adult T-cell leukemia (ATL) [1-4], in 2 to 5 % of infected individuals. HTLV-1 is also associated with chronic inflammatory diseases [5,6], including HTLV-1 associated myelopathy/tropical spastic paraparesis (HAM/TSP), uveitis, alveolitis [7], and dermatitis [8]. It has been estimated that 20 million people are infected with HTLV-1 in the world. HTLV-1 has a characteristic proliferative strategy; HTLV-1 increases its copy number not via vigorous production of cell-free viral particle but mainly via proliferation of infected host cells, which contain the integrated HTLV-1 provirus in the host genome [9,10]. Given the fact that HTLV-1 utilizes CD4^+^ T cells as the major host cell population, the pathogenesis by this virus may be due to abnormalities of CD4^+^ T cells in HTLV-1-infected individuals. However the precise characteristics of the putative CD4^+^ T-cell abnormality still remain to be elucidated.

In addition to viral structural proteins, such as Gag, Pol, and Env, HTLV-1 encodes several regulatory and accessory proteins, including Tax, Rex, p30, p12, and HTLV-1 bZIP factor (HBZ), which regulate viral gene expression or proliferation of infected host cells [4]. After the HTLV-1 provirus is integrated into the host genome, the virus expresses these regulatory and accessory proteins to induce host cell proliferation or viral latency, resulting in persistent infection *in vivo*. Tax is known to influence various host cell-signaling pathways, for example activation of NF-κB, and to contribute to proliferation and survival of infected cells [11,12]. Another viral gene, the HBZ, which is encoded in the minus strand of HTLV-1 [13] and expressed constitutively in the infected host cells [14,15], also contributes to proliferation of the infected cells [14,16], dysregulation of differentiation and function of CD4^+^ T cells [17], and the pathogenesis of diseases such as T-cell lymphoma and chronic inflammatory diseases [17,18]. On the other hand, viral protein expression induces the host immune response to eliminate the virus, which includes both antibody and cytotoxic T lymphocytes (CTL) against the viral antigens [19-21]. It has been reported that the CTL response against this virus determines HTLV-1 proviral load; yet, the host immune system cannot eliminate the HTLV-1 completely, which allows HTLV-1 to establish persistent infection in almost all infected individuals.

Recent studies have clarified the presence of various CD4^+^ T-cell subsets. CD4^+^ T cells can be divided into two major categories, effector T cells and regulatory T cells. Effector T cells induce the activation of immune responses by secreting pro-inflammatory cytokines whereas regulatory T cells, which express the transcription factor FoxP3 [22-24], suppress the immune response by both cell-contact dependent and independent mechanisms [25]. As an example of cell contact dependent suppression, expression of the immune suppressive molecule CTLA-4 on the cell surface inhibits the activation of surrounding neighboring T cells [26]. In addition, a recent report demonstrated that human FoxP3^+^CD4^+^ T cells were composed of three phenotypically and functionally different subsets according to the degree of FoxP3 expression and CD45RA expression, namely CD45RA^+^FoxP3^low^ resting T_reg_ cells (rT_reg_ cells), CD45RA^−^FoxP3^high^ activated T_reg_ cells (aT_reg_ cells), or CD45RA^−^FoxP3^low^ non-suppressive T cells (FoxP3^low^ non-Treg cells) [27]. Both rT_reg_ cells and aT_reg_ cells have suppressive function, but FoxP3^low^ non-Treg cells are not suppressive.

Previous studies have reported that the HTLV-1 provirus is enriched in effector/memory T cells [28,29], and the phenotype of ATL cells shares certain characteristics with regulatory T cells based on the finding of FoxP3 expression [30,31]. However there are few studies that systematically and specifically investigate which recently described CD4^+^ T-cell subset is infected by HTLV-1 in asymptomatic carriers (AC), HAM/TSP patients, and ATL patients. To elucidate this point, we analyzed peripheral mononuclear cells (PBMCs) from naturally HTLV-1-infected individuals, including AC, HAM/TSP, and ATL patients, by using multicolor flow cytometric analysis combined with the detection of the viral antigen Tax to identify the presence of HTLV-1 [32]. We found the specific CD4^+^FoxP3^+^ T-cell subset is frequently infected with HTLV-1, which may allow the virus to achieve persistent infection *in vivo*, and should also contribute to the pathogenesis of the virus-associated diseases.

## Results

### The frequency of FoxP3^+^ cells is positively correlated with HTLV-1 proviral load

**Figure 1:**
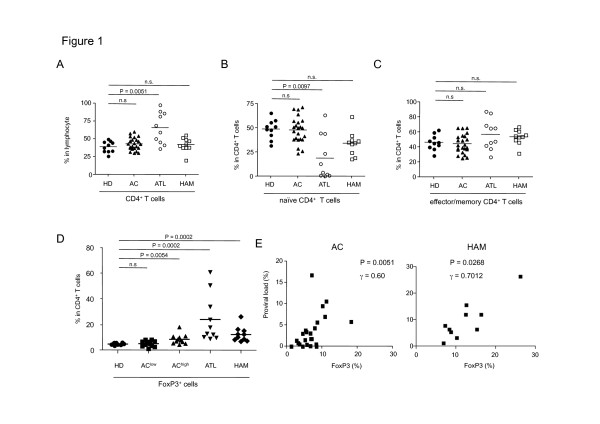
**CD4**^**+**^**T-cell subset in HTLV-1 infected individuals. (A)** Percentages of CD4^+^ T cells in 4 distinct subjects. Data shown are gated on lymphocyte fraction based on the dot plot pattern of SSC and FSC. **(B and C)** Proportion of FoxP3^−^CD45RA^+^ naïve CD4^+^ T cells (B) or FoxP3^−^CD45RA^−^ effector/memory CD4^+^ T cells (C). **(D)** Percentages of FoxP3^+^ cells in CD4^+^ T cells. **(E)** Frequency of FoxP3^+^CD4^+^ cells in ACs or HAM/TSP patients showed significant correlation with HTLV-1 proviral load by Spearman’s rank correlation (*P* = 0.0051 or *P* = 0.0268, r = 0.60 or r = 0.7012, respectively).

Previous studies reported that the HTLV-1 provirus was frequently detected in effector/memory CD4^+^ T cells [28], but at that time the analysis did not distinguish between effector/memory CD4^+^ T cell and regulatory T cells (T_reg_ cells). Also further subsets of CD4^+^ T cells have been identified recently, such as the division of FoxP3^+^CD4^+^ T cells into three distinct subsets [27]. In order to uncover the impact of HTLV-1 infection on the CD4^+^ T-cell subset, it is necessary to re-evaluate the CD4^+^ subsets in HTLV-1-infected individuals. We analyzed 23 ACs, 10 HAM/TSP patients, 10 ATL patients, and 10 healthy donors in this study as shown in Table 1. Almost all ATL cells express CD4, and indeed the percentage of CD4^+^ T-cells in ATL patients was significantly higher than that of uninfected healthy donors (*p* = 0.0051, Figure 1A). There were no significant differences in the percentage of CD4^+^ T cells between HD, AC, and HAM/TSP individuals (*p* = 0.2153 and 0.4597, respectively, Figure 1A). To characterize the CD4^+^ T-cell subset in more detail, we stained PBMCs with anti-CD4, anti-CD45RA, and anti-FoxP3 antibodies. In this analysis we divided CD4^+^ T cells into three distinct subsets, which include two FoxP3^−^ populations (CD45RA^+^ naïve T cells and CD45RA^−^ effector/memory T cells) and a FoxP3^+^ population. As shown in Figure 1B, the percentage of naïve CD4^+^ T cells was decreased in ATL patients (*p* = 0.0097), but did not differ significantly between HD, AC and HAM/TSP (*p* = 0.8381 and 0.2567, respectively). The percentages of effector/memory CD4^+^ T cells were not significantly different among the four studied subject groups (Figure 1C). However, frequencies of FoxP3^+^ cells in HTLV-1 infected individuals (AC^high^, ATL, HAM/TSP) were remarkably higher than those of HD (*p* = 0.0054, 0.0002 and 0.0002, respectively, Figure 1D). The frequencies of FoxP3^+^ cells in AC were significantly correlated with HTLV-1 proviral load (PVL) (r = 0.60, *p* = 0.0051, Figure 1E). Additionally, the absolute number of each T-cell subset showed the same tendency as well as the frequency (Additional file 1: Figure S1). These results collectively suggested that HTLV-1 infection increased the frequency of FoxP3^+^CD4^+^ T cells.

**Table 1:** Characteristics of participants

Characteristics	HD	AC	ATL	HAM/TSP
Participant number	10	23	10	10
Age, median years (IQR)	54 (49–62)	59 (50–70)	65 (61–76)	60 (54–62)
Male sex, no (%)	3 (30)	6 (26)	5 (50)	3 (30)
WBC (IQR)/μL	4,930 (1,437)	5,157 (1,100)	17,030 (12,975)	5,900 (1,500)
Lymphocyte (IQR)/μL	1,717 (503)	1,697 (601)	8,443 (10,764)	1,739 (560)
PVL median (IQR)	-	1.8 (0.5–5.0)	59.6 (18.3–67.1)	9.6 (5.6–12.0)

NOTE. *HD* healthy donor; *AC* asymptomatic HTLV-1 carrier; *ATL* adult T-cell leukemia; *HAM/TSP* HTLV-1 associated myelopathy/tropic spastic paraparesis; *IQR* interquartile range; *PVL* proviral load. ATL patients consist of 2 acute, 4 smoldering and 4 chronic types of ATL cases.

### Tax expression after *ex vivo* culture is well correlated with proviral load

**Figure 2:**
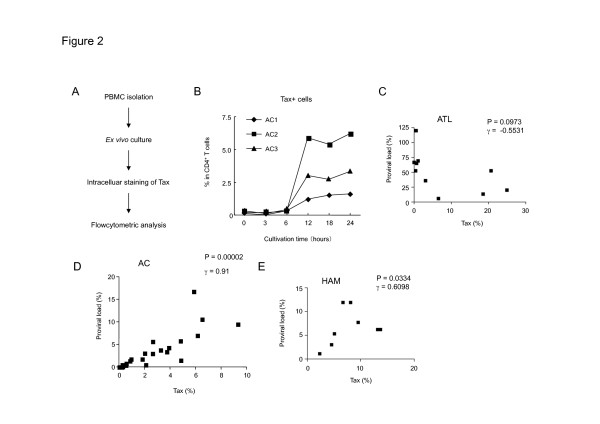
**Characterization of Tax expression after*ex vivo*cultivation. (A)** A flowchart of the experiment to detect Tax by flow cytometry. **(B)** The percentages of Tax expression in CD4^+^ T cells after *ex vivo* culture are shown from 3 distinct ACs. **(C–E)** Correlation between Tax positivity in CD4^+^ T cells and PVL in ATL patients (C), ACs (D) or HAM/TSP (E).

It has been reported that Tax expression increases spontaneously during *ex vivo* cultivation [32], which is useful to detect HTLV-1 infected cells at single cell level. We, therefore, used the same method to detect HTLV-1 infected cells by flow cytometry (Figure 2A), in which we can detect both Tax and various markers of CD4^+^ T-cell subsets at the same time. We first evaluated the detection system by using a series of samples collected at different time points after *ex vivo* cultivation. We found that a small number of Tax-expressing cells could be detected after *ex vivo* cultivation for 6 hours; significant expression could be observed after 12 hours cultivation; and Tax expression continued for 24 hours of cultivation (Figure 2B). In order to confirm the efficiency of this system, we analyzed the correlation between HTLV-1 proviral load and the percentage of Tax expression in this system.

Consistent with previous reports that Tax expression is frequently silenced in ATL cells, Tax expression after *ex vivo* cultivation of ATL cells was not correlated with the proviral load (Figure 2C). The percentage of Tax positive cells tended to be lower than the proviral load even after *ex vivo* culture in AC and HAM/TSP patients, but we found that Tax positivity showed a significant correlation with the proviral load both in AC and HAM/TSP (r = 0.91 or 0.61, *p* = 0.00002 or 0.0334, respectively, Figure 2D and E). In order to investigate whether T-cell subset markers, including FoxP3 and CD45RA, are influenced by *ex vivo* cultivation, we analyzed their expression both before and after cultivation. The results showed that the frequency of FoxP3 or CD45RA was not significantly changed during *ex vivo* culture (Additional file 2: Figure S2). These findings collectively indicate the usefulness of this Tax detection system for this study.

### The frequency of HTLV-1 infection in each CD4^+^ T-cell subset

**Figure 3:**
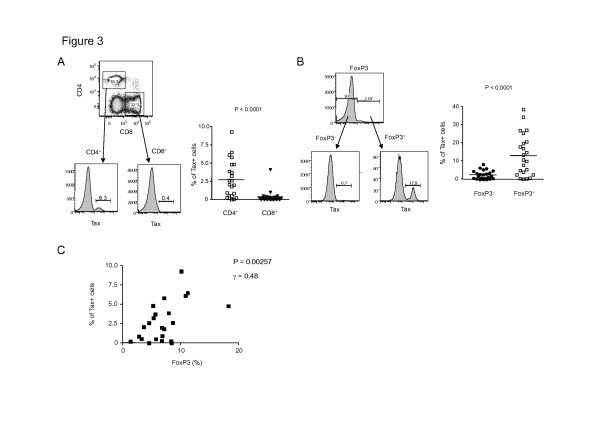
**Frequency of HTLV-1-infection in each CD4**^**+**^**T-cell subset of asymptomatic HTLV-1 carriers.** PBMCs from HTLV-1 asymptomatic carriers (n = 23) were cultivated for 18 hours, stained with anti-CD4, anti-CD8, anti-FoxP3, and anti-Tax antibodies, and analyzed by flow cytometry. **(A)** Representative dot plots of CD4 and CD8 and histograms of Tax in CD4^+^ or CD8^+^ T cells (Left panel). Right, cumulative results from 23 AC individuals are shown in graph (Right panel). **(B)** Representative histograms of Tax expression in FoxP3^+^ or FoxP3^−^ cell (Left panel). Right, cumulative results from 23 AC individuals are shown in graph (Right panel). **(C)** Tax positivity in CD4^+^ T cells showed significant correlation with FoxP3 positivity in CD4^+^ T cells by Spearman’s rank correlation (*P* = 0.0257, r = 0.48)

We next investigated which T-cell subset is frequently infected with HTLV-1. We cultivated PBMCs isolated from HTLV-1 infected individuals *ex vivo* for 12–18 hours and stained with antibodies to Tax and various T-cell subset markers such as CD4, CD8, and FoxP3. Consistent with the previous reports, the frequency of Tax positivity in CD4^+^ T cells was much higher than that in CD8^+^ T cells (*p* < 0.0001, Figure 3A). Among CD4^+^ T cells, the FoxP3 positive cell population contained a significantly higher ratio of Tax positive cells than that in FoxP3 negative cells (*p* < 0.0001, Figure 3B). In line with the finding in Figure 1E, the frequencies of FoxP3^+^ cells were significantly correlated with Tax positivity in CD4^+^ T cells. (r = 0.48, *p* = 0.0257, Figure 3C). These results indicated that the increased FoxP3^+^ cells in HTLV-1-infected individuals were frequently infected with HTLV-1.

### Characterization of FoxP3^+^CD4^+^ T-cell subset in AC

**Figure 4:**
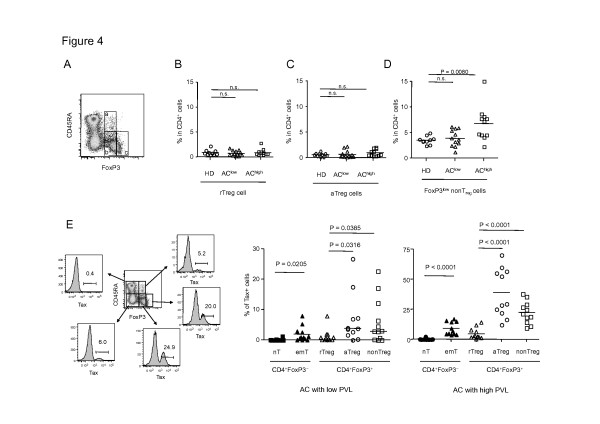
**Characterization of FoxP3**^**+**^**CD4**^**+**^**T-cell subset in AC samples. (A)** A result of flow cytometric dot plots of CD45RA and FoxP3 in CD4^+^ T cells of an AC sample is shown as an example. FoxP3^+^CD4^+^ T cells were classified into three subsets. a, CD45RA^+^FoxP3^low^ resting T_reg_ cells (rT_reg_ cells); b, CD45RA^−^FoxP3^low^ non-regulatory T cells (FoxP3^low^ non-T_reg_ cells), c, CD45RA^−^FoxP3^high^ activated T_reg_ cells (aT_reg_ cells). **(B–D)** Frequencies of the FoxP3^+^CD4^+^ T-cell subset in HD, AC^low^, and AC^high^. Percentages of resting T_reg_ cells (B), activated T_reg_ cells (C), and FoxP3^low^ non-T_reg_ cells (D) in CD4^+^ T cells are shown. **(E)** Representative flow cytometric dot plots of CD45RA and FoxP3 and histograms of Tax expression in each CD4^+^ T-cell subset in an AC sample (Left panel). Cumulative results from 13 AC^low^ and 10 AC^high^ individuals are shown in the graph (Middle and right panels).

We further focused on the FoxP3^+^CD4^+^ T-cell subset as defined previously (Figure 4A) [27]. First, we investigated the frequency of FoxP3^+^CD4^+^ T-cell subset in HD or AC. The results showed that the frequencies of rT_reg_ or aT_reg_ in AC^low^ or AC^high^ were not significantly different from that in HD (Figure 4B and 4 C), but FoxP3^low^ non-T_reg_ cells were significantly more frequent in the AC^high^ population (*p* = 0.0080, Figure 4D). We next analyzed the presence of HTLV-1 in each CD4^+^ T-cell subset by using AC sample. We observed that Tax positivity in FoxP3^−^ effector/memory CD4^+^ T cells was higher than that of FoxP3^−^ naïve CD4^+^ T cells (*p* < 0.0001, Figure 4E). Since effector/memory CD4 T cells are the most dominant in total CD4 T cells in terms of absolute cell number, the Tax-expressing cells are most abundant in effector/memory CD4 T cells (Additional file 3: Figure S3). More interestingly, Tax positivity in aT_reg_ cells or FoxP3^low^ non-T_reg_ cells was much higher than that of rT_reg_ cells in ACboth AC^low^ and AC^high^ subjects (*p* < 0.0001 or 0.0001, respectively, Figure 4E). These results indicated that HTLV-1 is frequently present in aT_reg_ cells or FoxP3^low^ non-T_reg_ cells.

### Characteristics of T-cell subsets in HAM/TSP patients

**Figure 5:**
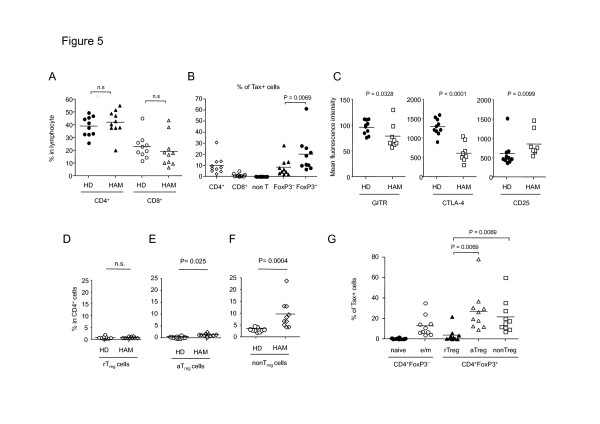
**Detailed characterizations of CD4**^**+**^**T-cell subsets in HAM/TSP patients.** Freshly isolated PBMCs from HAM/TSP patients (n = 10) were stained with anti-CD4, anti-CD45RA, anti-FoxP3 antibodies and analyzed by flow cytometry. To detect Tax expression, PBMCs were cultivated for 18 hours before antibody staining. **(A)** Percentages of CD4^+^ or CD8^+^ T cells in HAM/TSP patients. **(B)** Tax positivity of CD4^+^, CD8^+^, non-T, FoxP3^−^, or FoxP3^+^ cell populations in HAM/TSP patients. **(C)** Expression levels of T_reg_ associated molecules in FoxP3^+^ cells of HD or HAM/TSP patients. **(D–F)** Frequencies of the FoxP3^+^CD4^+^ T-cell subset in HD and HAM/TSP patients. Percentages of rT_reg_ cells (D), aT_reg_ cells (E), and FoxP3^low^ non-T_reg_ cells (F) in CD4^+^ T cells are shown. **(G)** Tax positivity of each CD4^+^ T-cell subset in HAM/TSP patients

To investigate the inflammatory aspects of HTLV-1 infection, we next focused on PBMCs of HAM/TSP patients. There were no significant differences in the percentage of CD4^+^ or CD8^+^ T cells between HD and HAM/TSP groups (*p* = 0.3073 and 0.1509, respectively, Figure 5A). The result of Tax staining showed that HTLV-1 infection was predominantly detected in CD4^+^ T cells, and at a higher frequency in CD4^+^FoxP3^+^ T cells than CD4^+^FoxP3^−^ T cells (*p* = 0.0069, Figure 5B). To characterize the phenotype of FoxP3^+^ cells in HAM/TSP patients, we investigated the expression levels of T_reg_ associated molecules, and found that the expression of GITR or CTLA-4 in HAM/TSP patients was significantly lower than that in HD (*p* = 0.0328 or 0.00002, respectively, Figure 5C). On the contrary, CD25 expression was high in HAM/TSP patients (*p* = 0.0099, Figure 5C). We further evaluated FoxP3^+^CD4^+^ T-cell subset in HAM/TSP patients. The frequencies of rT_reg_ were not significantly different from that in HD (*p* =0.9096, Figure 5D), but aT_reg_ cells or FoxP3^low^ non-T_reg_ cells were remarkably increased (*p* = 0.0250 or 0.0004, Figure 5E and 5F). Furthermore, aT_reg_ cells or FoxP3^low^ non-T_reg_ cells showed a high frequency of Tax + cells compared with rT_reg_ cells (*p* = 0.0069 or 0.0069, respectively, Figure 5G) as observed in ACs (Figure 4E). These data indicated that HTLV-1 infection significantly influenced not only the frequency but also the phenotype of CD4^+^FoxP3^+^ T cells in an inflammatory disease HAM/TSP.

### Phenotypical analyses of ATL cells

**Figure 6:**
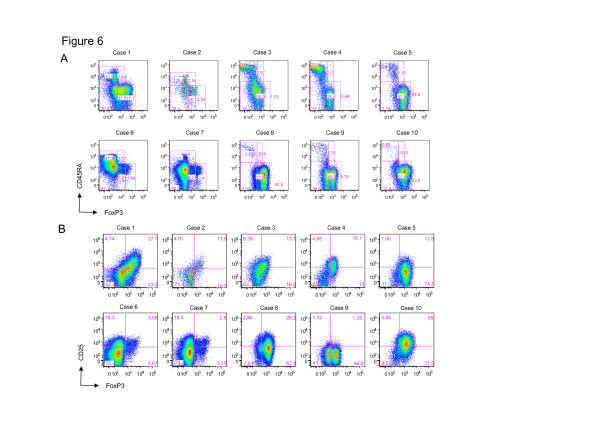
**Characterization of CD4**^**+**^**T-cell subsets in ATL cells.** PBMCs from ATL patients (n = 10) were stained with anti-CD4, anti-CD45RA, anti-CD25, and anti-FoxP3 antibodies and analyzed by flow cytometry. **(A)** The expression pattern of CD45RA and FoxP3 of CD4^+^ T cells in ATL patients. **(B)** The expression pattern of CD25 and FoxP3 of CD4^+^ T cells in ATL patients.

Previous studies reported that some ATL cells express FoxP3 or CD25 [30,31,33], but the precise information about FoxP3^+^CD4^+^ T-cell subset of ATL cells remains unknown. We, therefore, analyzed CD4^+^ T-cell subsets for ATL cases. FoxP3 positivity was 80% in ATL cases; yet the expression level was different among the cases (Figure 6A), which is consistent with previous reports [30,31]. In line with the finding in asymptomatic HTLV-1-infected carriers that the percentage of HTLV-1 in FoxP3^low^ non-T_reg_ cells or aT_reg_ cells was high (Figure 4E), ATL cells analyzed in this study did not express CD45RA, suggesting that FoxP3-expressing ATL cells might be derived from FoxP3^low^ non-T_reg_ or aT_reg_ cells. CD25 expression on ATL cells was generally high, but there was also much variation among the cases (Figure 6B).

## Discussion

FoxP3^+^ T_reg_ cells play a crucial role in persistent infection and pathogenesis of chronic viral infection. Previous studies have suggested that T_reg_ cells suppress virus-specific CD8^+^ T-cell effector functions in chronic human viral infections such as human immunodeficiency virus, hepatitis C virus and cytomegalovirus [34,35]. Regarding this point, FoxP3^+^ T_reg_ cells play a role in facilitating viral persistence. In HTLV-1 infection, the frequency of FoxP3^+^ cells is indeed correlated with the impairment of CTL activity against the viral antigen Tax in HAM/TSP patient [36]. On the other hand, FoxP3^+^ T_reg_ cells could prevent tissue damage caused by excessive immune response triggered by viral infection. In addition to these general roles of FoxP3^+^ T_reg_ cells in chronic viral infection, FoxP3^+^ T_reg_ cells should have some specific role in HTLV-1 infection, because FoxP3^+^ T_reg_ cells are comprised in CD4^+^ T cells, which are a main host cell population of HTLV-1. Here we performed a comprehensive analysis of CD4^+^ T-cell subsets in individuals naturally infected with HTLV-1 and revealed that the frequency of HTLV-1 infection is positively correlated with the frequency of FoxP3^+^ T cells (Figure 1E). The increased FoxP3^+^ T cells themselves are frequently infected with HTLV-1 (Figure 3B), suggesting that HTLV-1 utilizes the FoxP3^+^ T cells as a host cell. What is the advantage for HTLV-1 to exist in FoxP3^+^ T cells? There are two possibilities for this preference. First, FoxP3^+^ T cells are known as hyper-proliferating cells *in vivo* with a doubling time of 8 days [37], which could contribute to clonal expansion of infected cells. Second, HTLV-1 can evade the host immune system by directly infecting this potentially immuno-suppressive cell population. Thus, HTLV-1-infection of FoxP3^+^ T cells should enable the virus to increase or maintain proviral load and achieve persistent infection.

How then does HTLV-1 infection target FoxP3^+^ T cells? This could be explained by the following two mechanisms. First, FoxP3^+^ T cells are known to contact with dendritic cells (DCs) frequently [38], which could increase the chance of *de novo* viral infection between DCs and FoxP3^+^ T cells. A recent study demonstrated that cell-free HTLV-1 efficiently infects DCs, and the infected DCs promote *de novo* infection of CD4^+^ T cells [39]. This notion is consistent with the finding that effector/memory-type CD45RA^−^ T_reg_ cells, including FoxP3^low^ non-T_reg_ cells and FoxP3^high^ aT_reg_ cells, are more frequently infected with HTLV-1 than CD45RA^+^ rT_reg_ cells (Figure 4E). Second, once FoxP3^−^ T cells are infected with HTLV-1, HBZ should be expressed in the host cells. Since HBZ is recently reported to induce FoxP3 expression via enhancing TGF-β signaling pathway [17,40], HTLV-1 infection is likely to convert FoxP3^−^ cells into FoxP3^+^ cells. In addition, HTLV-1 has a cell-extrinsic effect on FoxP3^+^ cell generation. HTLV-1 infected cells secrete CCL22 via expression of Tax, which indirectly contributes to the generation and maintenance of HTLV-1 uninfected FoxP3^+^ cells [41,42]. This would contribute to an increased number of HTLV-1-uninfected FoxP3^+^ cells.

Since FoxP3^+^ T_reg_ cells play a crucial role in suppressing immune response, the increase of FoxP3^+^ cells observed in HTLV-1 infection may contribute to immunodeficiency, which is frequently observed in HTLV-1 infection [43]. On the other hand, the high frequency of FoxP3^+^ T cells observed in HAM/TSP patients is paradoxical, because the pathogenesis of HAM/TSP is believed to be inflammatory. Therefore, we analyzed the phenotype of the increased FoxP3^+^ cells and observed that CTLA-4 and GITR expression of FoxP3^+^ T cells in HAM/TSP patient was significantly reduced compared to uninfected individuals (Figure 5C). A similar observation was reported previously that the expression level of FoxP3, GITR, or CTLA-4 mRNA in CD4^+^CD25^+^ T cells of HAM/TSP patients is lower than that of HD [44]. That report used CD4^+^CD25^+^ as a marker of T_reg_ cells, but CD4^+^CD25^+^ T cells contain not only FoxP3^+^ T_reg_ cells but also FoxP3^−^ activated T cells. Particularly the proportion of CD4^+^CD25^+^FoxP3^−^activated T cells is up-regulated in HAM/TSP patients, which is likely to reduce the proportion of FoxP3^+^ T_reg_ cells in CD4^+^CD25^+^ T cells of HAM/TSP patients. Thus, the expression level of GITR or CTLA-4 in FoxP3^+^ T cells of HAM/TSP patients has not been elucidated yet. To avoid this concern, we utilized the multicolor flow cytometry, which enabled us to show that CTLA-4 and GITR were clearly down regulated in FoxP3^+^ T cells of HAM/TSP patients.

Then what is the underlying mechanism of this phenomenon? We reported recently that HBZ-Tg mice showed a pro-inflammatory phenotype in spite of the increase of Foxp3^+^ T cells [17], which is similar to HAM/TSP patients (Figure 1D). T_reg_ associated molecules were also down regulated in Foxp3^+^ T cells of HBZ-Tg mice. Thus, HBZ-mediated FoxP3 dysfunction may play a role in the abnormality regarding FoxP3^+^ cells in HAM/TSP patients. It has been reported that Tax also contributes to the dysregulation of FoxP3^+^ T_reg_ cells. Tax suppresses FoxP3 expression at transcriptional level [45], which alternatively or additionally could contribute to the abnormal phenotype of FoxP3^+^ cells. These findings collectively indicate that the increased FoxP3^+^ T_reg_ cells were functionally impaired in HAM/TSP patients. Furthermore, FoxP3^+^CD4^+^ T cells in HAM/TSP patient contain an increased FoxP3^+^ non-Treg population (Figure 5F), which would contribute to the inflammatory phenotype of HAM/TSP via generation of pro-inflammatory cytokine-producing CD4^+^ T cells such as T_HAM_ cells [46] or exFoxp3 cells [47].

In the current study, we did not observed FoxP3 repression during Tax expression by *ex vivo* cultivation. This result seems to be inconsistent with a previous report that Tax represses FoxP3 expression [45]. There are two possible explanation of this inconsistency. First, there is the difference of the ways to express Tax. In the previous study, the authors used transfection of plasmid that induces Tax expression by the CMV promoter. We used endogenous HTLV-1 provirus to express Tax. Therefore, the expression level of Tax in our current study should be much lower than that of the previous study. In addition, Tax expression was induced in a proportion of FoxP3^+^ cell in our current study. Second, there are differences in incubation time for Tax expression. In the previous study, the authors evaluated FoxP3 expression after 48 hours of transfection, whereas we evaluated FoxP3 expression within 24 hours after Tax expression.

High expression levels of CD25 are also well documented in HTLV-1 infection [33]. Consistent with previous findings, CD25 expression is upregulated in FoxP3^+^ cells of HAM/TSP patient (Figure 5C). One determinant of the susceptibility to HAM/TSP is host genetic polymorphism such as MHC class 1, which influences the efficiency of CTL against HTLV-1 [48,49]. HTLV-1-infected individuals who have HLA class I susceptible for HAM/TSP may allow high expression of Tax and/or HBZ, which could cause up-regulation of CD25 molecules in the FoxP3^+^ cell population (Figure 5C).

It is controversial whether ATL is a leukemia of FoxP3^+^ T_reg_ cells or not. However, there is no *a priori* reason to assume that ATL cells must be exclusively derived from FoxP3^+^ T_reg_ cells or non-T_reg_ cells. Indeed, there are previous reports to support both possibilities. Some studies have reported that ATL cells have regulatory functions [50,51], whereas other studies reported no regulatory function in ATL [52,53]. We showed here that HTLV-1 is frequently detectable in CD4^+^FoxP3^+^ T cells (Figure 3B) in AC. More than half of ATL cells express FoxP3 (Figure 6), even though FoxP3 expression in ATL cells is variable as shown in the present and previous studies [30,31]. These findings prompt us to propose an idea that more than a half of ATL cells are possibly derived from FoxP3^+^ T_reg_ cells. We reported previously that HBZ expression is constitutively active but Tax expression is frequently silenced in ATL cells, which possibly contributes to high frequency of FoxP3^+^ ATL.

## Conclusion

This study demonstrated that HTLV-1 infection induced the abnormality of frequency and phenotype of FoxP3^+^ T cells, suggesting that HTLV-1 has evolved a sophisticated strategy to achieve persistent infection by directly affecting the central regulator of the host immune system. HTLV-1-mediated dysregulation of FoxP3^+^ T cells is likely to be a critical cellular mechanism for the understanding HTLV-1 pathogenicity.

## Methods

### Clinical samples and ethics statement

PBMCs were obtained from asymptomatic HTLV-1 infected carriers (n = 23), HAM/TSP patients (n = 10), ATL patients (n = 10), and age-matched healthy controls (n = 10). Characteristics of each group are presented in Table 1. ATL patients consist of 2 acute, 4 smoldering and 4 chronic types of ATL cases. Genomic DNA extracted from PBMCs was used to determine proviral load (PVL) as described previously [29]. Briefly, PVL was quantified by real time PCR and calculated by using genomic DNA of TL-Om1, an ATL cell line with one copy of complete HTLV-1 provirus, as a standard of 100%. We defined AC with less than 2% of proviral load as AC^low^ and AC with more than 2% of proviral load as AC^high^. This study was conducted according to the principles expressed in the Declaration of Helsinki and approved by the Institutional Review Board of Kyoto University (844 and E-921). All patients provided written informed consent for the collection of samples and subsequent analysis.

### Antibodies

The following antibodies were purchased from BD PharMingen; purified monoclonal antibody (mAb) for human CD3 (UCHT1), CD4 (RPA-T4), CD8a (RPA-T8), CD45RA (NI100) and CTLA-4 (BNI3). Purified mAbs for human CD25 (BC96), GITR (eBio AITR) and FoxP3 (236A/E7) were purchased from eBioscience.

### Flow cytometric analysis

PBMCs were isolated with Ficoll-Isopaque (GE Healthcare) gradient centrifugation. Flow cytometric analyses were carried out using a FACS CantoII with Diva Software (BD Pharmingen), and the data were analyzed by FlowJo software (Treestar). To discriminate dead cells, we used LIVE/DEAD Fixable Near-IR Dead Cell Stain Kit (Invitrogen). For cell surface staining, 10^6^ cells were incubated with mAbs for 30 minutes at 4°C, and then analyzed. For intracellular staining, we used a human FoxP3 staining kit according to the manufacture’s protocol (eBioscience). To distinguish FoxP3^+^ and FoxP3^−^ cell population clearly, we used isotype control according to the manufacture’s recommendation. To detect the viral antigen Tax, we cultured PBMCs from ACs or HAM/TSP patients for 12–18 hours and stained with monoclonal antibodies against FoxP3 or Tax (MI-73) [54], and then analyzed by flow cytometry.

### Statistical analysis

To compare 2 groups when data were determined to have a Gaussian distribution, the Student *t* test was used. If data did not have a Gaussian distribution, the Mann–Whitney *U* test was used for unpaired data, and the Wilcox signed-ranks test was used for paired data. The AC group and HD did not differ significantly in sex or age, using chi-squared test and Mann–Whitney *U* test. Differences with *P* < 0.05 were considered to be statistically significant. Correlations were evaluated using Spearman’s rank correlation.

## Competing interests

The authors declare that they have no competing interests.

## Authors’ contributions

This study was designed by YS and MM. Laboratory analysis was performed by YS and JT. Data analysis was performed by YS, AU, JT and MM. Clinical samples and data were provided by AU, MN and KN. YS and MM wrote the paper. All authors read and approved the final manuscript.

## Supplementary Material

Additional file 1: Figure S1. Absolute cell numbers of each CD4^+^T-cell subset in HTLV-1 infected individuals. (A) Absolute cell numbers of CD4^+^ T cells in 4 distinct subjects. Data shown are gated on lymphocyte fraction based on the dot plot pattern of SSC and FSC. (B and C) Absolute cell numbers of FoxP3^−^CD45RA^+^ naïve CD4^+^ T cells (B) or FoxP3^−^CD45RA^−^ effector/memory CD4^+^ T cells (C). (D) Absolute cell numbers of FoxP3^+^ cells in CD4^+^ T cells. Click here for file

Additional file 2: Figure S2. Effect of *ex vivo* cultivation on FoxP3 and CD45RA expression. The percentages of FoxP3 and CD45RA expression in CD4^+^ T cells both before and after *ex vivo* culture are shown from 5 distinct ACs. Click here for file

Additional file 3: Figure S3. Frequency of each CD4 T-cell subset in Tax-expressing cell population in AC. Cumulative results from 23AC individuals are shown in the graph. Click here for file
