# Supplementary figures and images for: HTLV-1 modulates the frequency and phenotype of FoxP3+CD4+ T cells in virus-infected individuals
Source: Retrovirology. 2012 May 30;9:46. doi: 10.1186/1742-4690-9-46 (PMC3403885; doi:10.1186/1742-4690-9-46)

Figure S1

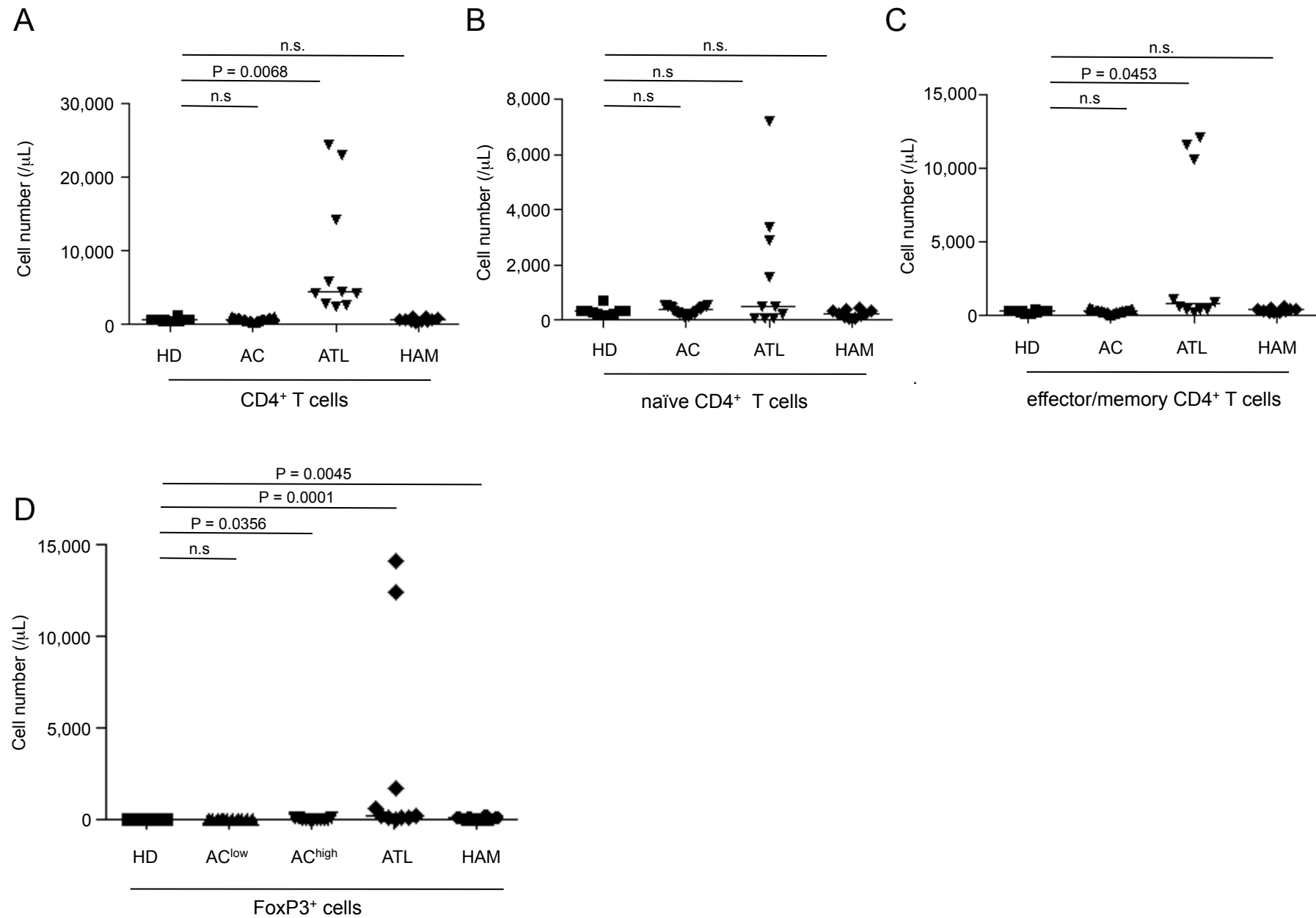

Supplement: Additional file 1: Figure S1. — Absolute cell numbers of each CD4+T-cell subset in HTLV-1 infected individuals. (A) Absolute cell numbers of CD4+ T cells in 4 distinct subjects. Data shown are gated on lymphocyte fraction based on the dot plot pattern of SSC and FSC. (B and C) Absolute cell numbers of FoxP3−CD45RA+ naïve CD4+ T cells (B) or FoxP3−CD45RA− effector/memory CD4+ T cells (C). (D) Absolute cell numbers of FoxP3+ cells in CD4+ T cells. [file 1742-4690-9-46-S1.pdf]

Figure S2

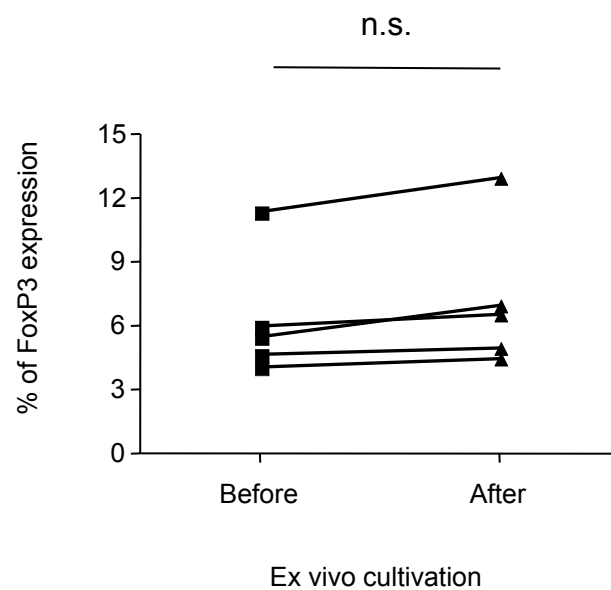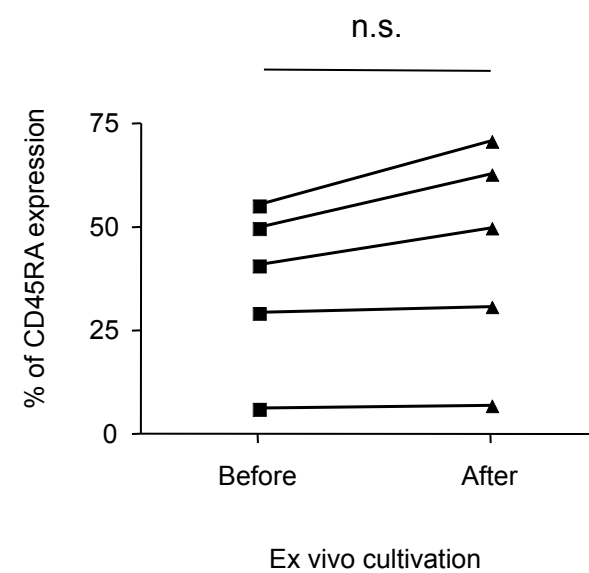

Supplement: Additional file 2: Figure S2. — Effect of ex vivo cultivation on FoxP3 and CD45RA expression. The percentages of FoxP3 and CD45RA expression in CD4+ T cells both before and after ex vivo culture are shown from 5 distinct ACs. [file 1742-4690-9-46-S2.pdf]

Figure S3

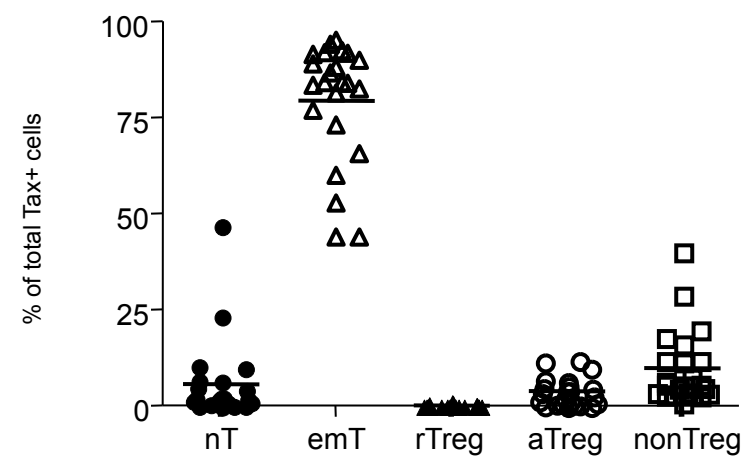

Supplement: Additional file 3: Figure S3. — Frequency of each CD4 T-cell subset in Tax-expressing cell population in AC. Cumulative results from 23AC individuals are shown in the graph. [file 1742-4690-9-46-S3.pdf]
